# Modern Treatment of Alcoholism and Drug Narcotism

**Published:** 1911-04

**Authors:** 


**Modern Treatment of Alcoholism and Drug Narcotism.** By **C. A. McBride,** M.D., London. 12 mo, pp. 384. New York: Rebman Co. (Cloth, $2.00.)

This most excellent work summarises the practical experience obtained from 30 years treatment of alcoholics and habitués.

After defining his terms and discussing the pathology of these affections, the etiology is fully dealt with. McBride considers the rôle played by heredity, environment, religious observance, worry, trouble and disease. The diseases that are contributing factors are, cardiac conditions, indigestion, neurasthenia and other nervous states, syphilis and mental conditions. Considerable attention is given to social custom and the baneful results of the treating habit are cogently discussed.

In the classification of the forms of inebriety, the author does not adopt the complicated division and subdivision of his predecessors but justly recognises the simple grouping into fine types of drink habitués; (1) the constant drinker, (2 the periodical drinker, (3) the dipsomaniac, (4) the voluntary drinker and (5) the mixed cases.

Chapter V, by far the most important of the work, covers 140 pages and discusses the diagnosis and treatment of this worldwide affection. The author strongly and rightly opposes the consideration of inebriety as a crime and regards imprisonment at best a temporary makeshift, for after sobering up the drunk usually reverts to his former state. The moral treatment, generally little used by the average practitioner, is amply discussed and advised in all cases. In the last analysis education is a factor with the alcholic as with other habitués.

In considering diet as a remedial agent, "the Saturation cure of the Luschs" is strongly condemned. The author favors a generous mixed diet with a preponderance of fruit and vegetables and uses these as an adjunct to his treatment rather than a curaative agent advocated by some authorities. The value of traveling, restraint, and colonisation of chronic alcholics is discussed and advised in properly selected cases.

After thirty years study and experience, McBride has devised a method of treatment by drugs successfully used in his sanitarium. It covers a period of 6 weeks for the average case and consists of hypodermic injection t. i. d. of strychnine nitrate and atropine sulphate in gradually increasing dosage. Simultaneously the simple bitter tonics are given by mouth. Elimination is provided for by catharsis and serates. Alchol is not absolutely interdicted but is used for the first few days in the form of whiskey. Usually about the third day the patient loses the desire and in the large majority of cases becomes a teetotaler remaining so for his natural life. Delirium tremens treated by substituting morphine and hyoscine for atropia. While not so rapid in results as the "Lambert Treatment" so called it is, however, more effectual and reliable.

Inebriety due to ether, chloroform, tea, coffee, opium and cocaine are fully discussed and treatment carefully planned.

The book is well written. It abounds in a large number of selected cases taken from actual experience, does not advance any obnoxious or repulsive theories but is rather interesting as well as instructive. It deserves careful reading and consideration from the general practitioner as well as the specialist.

